# A prospective randomized comparative placebo-controlled double-blind study in two groups to assess the effect of the use of biologically active additives with Siberian fir terpenes for the biological age of a person

**DOI:** 10.3389/fphar.2023.1150504

**Published:** 2023-03-02

**Authors:** Faniya Maganova, Mikhail Voevoda, Vladimir Popov, Alexey Moskalev

**Affiliations:** ^1^ Initium-Pharm LLC, Moscow, Russia; ^2^ Federal Research Center of Fundamental and Transnational Medicine, Moscow, Russia; ^3^ Department of Internal Medicine with a Pharmacy Course of the Medical Institute of Continuing Education, Federal State Budgetary Educational Institution of Higher Education Russian Biotechnological University, Moscow, Russia; ^4^ Department of Biochemistry and Pharmacology at Medical Institute of Tambov State University Named After G.R. Derzhavin, Tambov, Russia; ^5^ Laboratory of Genetics and Epigenetics of Aging, Russian Clinical and Research Center of Gerontology, Pirogov Russian National Research Medical University, Moscow, Russia; ^6^ Institute of Biogerontology, Lobachevsky State University of Nizhny Novgorod, Nizhny Novgorod, Russia

**Keywords:** terpenes, dietary supplement, artery stiffness, pulse wave velocity, carotid intima-media thickness, biological age, ultrasound, applanation tonometry

## Abstract

A prospective randomized comparative placebo-controlled double-blind study was carried out based on Arterial Indices model of biological age. The study involved 60 men and women aged 40–65 years that were randomly divided into two equal groups of 30 people: the main group and the control one. The study participants from the main group received a dietary supplement containing Siberian fir terpenes, limonene, alpha-linolenic acid, and vitamin E—1 capsule 3 times a day for 90 days. Patients in the comparison group received a placebo according to a similar scheme. Anthropometric and biochemical characteristics of patients from both groups have not undergone any significant changes. According to ultrasound examination of the carotid arteries, we observed a statistically significant decrease in the minimum thickness of the intima-media complex (by 45%). The maximum carotid artery stenosis on the right or left and the expansion index in patients of both groups did not change significantly during treatment. According to the results of applanation tonometry, it was revealed that when taking the studied dietary supplement, the pulse wave velocity significantly decreased compared to the initial one (by 10%). Accordingly, the Arterial Indices biological age decreased by 2.5 years compared to the baseline level in patients of the main group and did not change in patients from the comparison group. Supplementation of fir terpenes in middle-aged patients of both sexes reduces the biological age reflecting the condition of the arteries.

## Introduction

The concept of biological age appeared because of the awareness of the unevenness of aging (Moskalev, 2019). It is obvious that the intensity of aging is related to heredity, environmental conditions in the place of residence, the level of medical care and the lifestyle of the person. Therefore, with the same chronological age in different people, the degree of deterioration of the whole body, as well as individual organs and systems, is different. The consequences of age-related processes are also expressed to varying degrees—violations of the most important vital functions, narrowing of the range of adaptation, resilience, stress-resistance, development of disease states. We can assume that the difference between chronological and biological age reflects the intensity of aging and the risks of age-related diseases.

Considering the conventionality of the concept of biological age, researchers have made numerous attempts to establish a set of measurable criteria of aging. For various models of biological age, empirical clinical parameters (biochemical and functional), aging-based molecular measurements, or big omics data (methylome, transcriptome, proteome, metabolome, metagenome) are currently used (Moskalev, 2020).

An original method for determining the biological age has been proposed, based on the determination of sex-specific Arterial Indices model (Fedintsev et al., 2017). The method allows the use of widely used medical equipment in hospitals and clinics without performing molecular or cellular tests. Arterial indices are determined non-invasively by combining four functional indicators of cardiovascular health from the results of carotid duplex scanning and applanation tonometry.

Cardiovascular aging is characterized by a complex of pathophysiological changes affecting both the myocardium and blood vessel walls at the structural, cellular, molecular, and functional levels. As it is known, cardiovascular diseases are the main component of age-related mortality. Aging is associated with functional changes in blood vessels, including stiffening of the arteries, which is the main cause of hypertension. Moreover, a study in a recent publication has shown that arterial aging correlates better with chronological age than with the accompanying changes in blood biochemical parameters (Fedintsev et al., 2017). Carotid intima-media thickness (cIMT) is an established surrogate marker of atherosclerosis. This parameter is also associated with metabolic syndrome, insulin sensitivity and other age-related functional disorders.

**FIGURE 1:**
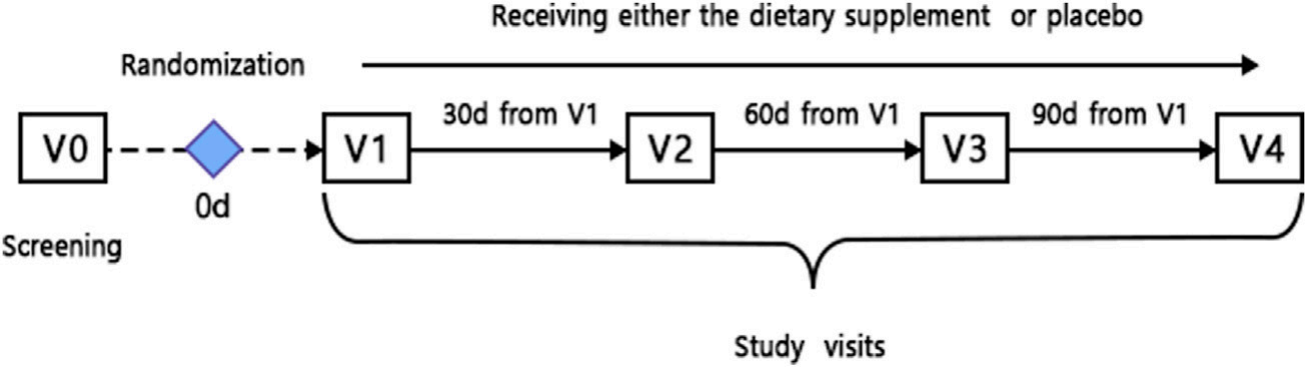
Schematic visit schedule.

The aim of this study was to investigate the effect of Siberian fir terpenes diet supplement in healthy middle-aged people. The impact of the study dietary supplement was assessed by the following primary endpoints—biological age determined by ultrasound and applanation tonometry (B4/B1) in healthy middle-aged subjects (Figure 1).

## Materials and methods

### Characteristics of patients participating in the study

A prospective, randomized, comparative, placebo-controlled, double-blind study was conducted based on N.I. cc, a separate structural subdivision of the Russian Gerontological Research and Clinical Center.

The study was conducted in accordance with the requirements of the Russian Federation National Standard “Good Clinical Practice” GOST R 53279–2005, the World Medical Association Declaration of Helsinki Ethical Principles for Medical Research Involving Human Subjects and ICH E6 Good Clinical Practice (GCP) rules and approved by the ethical committee of the Pirogov Russian State Medical University on 12/30/2020.

The study involved 60 men and women aged 40–65 years who signed an informed consent form.

The exclusion criteria were:

- The presence of any of the diseases or conditions: diabetes mellitus; body mass index (BMI) ≤ 25 or ≥ 38 kg/m2; arterial hypertension of the second or third degree; acute coronary syndrome or acute cerebrovascular accident or transient ischemic attack or revascularization interventions on coronary or brachiocephalic arteries in the anamnesis; atrial fibrillation; angina; chronic heart failure; GFR ≤ 59 ml/min/1.73 m2; increased activity of AST or ALT serum more than 2.5 times from the upper limit of the norm; chronic hepatitis or cirrhosis of the liver of any etiology; oncological disease of any localization at present or in the anamnesis.
- Current or previously conducted regular drug therapy, including all dietary supplements, antidiabetic drugs, statins, NSAIDs, RAAS blockers in less than 14 days or 5 half-lives.
- Hypersensitivity to the test product and/or its component in the anamnesis.
- Simultaneous participation in another clinical trial.
- Pregnancy, breastfeeding.
- A history of alcohol and/or drug addiction.

After the examination, the patients were randomly divided into two equal groups of 30 people: the main and the comparison group. Table 1 shows the anthropometric and biochemical characteristics of patients from both groups. Individual data collected in the Supplementary Table S1.

**TABLE 1:** Anthropometric and biochemical characteristics of patients from both groups.

Indicator	Comparison group	Main group	Reference values
Demographic and anthropometric characteristics			
Gender: men, number of persons women, number of persons	2	8	
	28	22	
Age,	45.5	51.0	
years	42.2–51.0	45.5–57.5	
Body mass index, kg/m^2^	31.2	30.1	18.5–25—norm
	28.1–34.0	28.6–31.8	25–30—overweight over 30 - obesity
Heart rate, beats per min	72.5	74.0	
	65.5–75.0	69.5–80.0	
Systolic blood pressure, mmHg	129	129	
	125–134	122–134	
Diastolic blood pressure, mmHg	82	82	
	76–90	77–90	
Markers of carbohydrate metabolism and insulin resistance			
Glycated hemoglobin,	5.50	5.50	4.27–6.07
%	5.35–5.70	5.40–5.65	
Fasting venous blood glucose, mmol/L	5.22	5.36	4.1–5.9
	5.08–5.39	5.12–5.55	
Insulin, mkU/ml	8.29	9.21	2.1–27
	5.68–12.17	5.75–13.45	
Markers of blood lipid profile			
Triglycerides, mmol/L	1.02	1.14	0.68–6
	0.82–1.57	0.90–2.08	
Cholesterol—HDL, mmol/L	1.58	1.60	< 3.3
	1.51–1.82	1.34–1.88	
Cholesterol—LDL, mmol/L	3.66	3.71	1.81–4.04
	3.14–4.33	3.21–4.60	
Markers of endothelial dysfunction			
The Willebrand Factor,	140	115.0	70–150
%	114–159	83.5–154.0	
Homocysteine, mmol/L	9.15	9.7	<20
	7.64–11.0	9.0–11.25	

Note: The data in the table are presented in the form of median and interquartile ranges.

Study participants from the main group received the dietary supplement “CardioOrganic®" 1 capsule 3 times a day, 20 min before meals for 90 days.

Patients in the comparison group according to a similar scheme received a placebo, which was a capsule of the same shape, color, and size as the study product, following a similar scheme.

### Medications used

Dietary supplement Vitaterpen brand “CardioOrganic®” produced by Korolevpharm LLC, contains biologically active substances of natural origin in an amount not exceeding the upper permissible level of consumption. One capsule (600 mg) contains at least: 20 mg of Siberian fir terpenes, 1.7 mg of limonene, 250 mg of omega-3 PUFA (alpha-linolenic acid), 6.5 mg of vitamin E. The quantitative composition of the placebo; 1 capsule (600 mg) contains at least: 592.5 mg - linseed oil, 7.5 mg—of vitamin E.

### Defined parameters

Primary endpoints of the clinical study—estimated indicators of biological age:

1. Biological age determined by ultrasound and applanation tonometry (B4/B1)
2. Biological age determined from the results of a blood test (B4/B1)

Secondary endpoints—additional indicators (blood):

1. Score of the quality of life according to the SF-36 questionnaire (B4/B1)
2. Interleukin-6 (B4/B1)
3. C-reactive protein (B4/B1)
4. TNF-α (B4/B1)
5. Ferritin (B4/B1)
6. Lipid peroxidation (B4/B1)
7. Insulin (B4/B1)
8. Willebrand factor (B4/B1)
9. Homocysteine (B4/B1)
10. Omega-3 index (B4/B1)
11. Unsaturated fatty acids (B4/B1)
12. Insulin-like growth factor IGF-1, (somatomedin C) (B4/B1)
13. Platelet aggregation with ADP (B4/B1)

In the dynamics of observation, a physical examination of patients, a biochemical blood test, Doppler ultrasound of the carotid arteries and applanation tonometry were performed; the frequency of adverse events and adverse reactions was noted, the value of biological age was calculated.

The pulse wave velocity was measured using the SphygmoCor device (AtCor Medical, Australia). The applanation tonometer is sequentially superimposed on the proximal (carotid) and, with a short interval, on the distal (femoral) artery, while an ECG is simultaneously recorded. The pulse wave velocity is calculated using the time of passage of the wave between the registration points, determined using the R wave on the ECG.

Duplex examination of the carotid arteries was performed using (Ultrasound Diagnostic Medical System, Vivid E9, Israel).

Biological age was calculated according to the formulas for women and men (Fedintsev et al., 2017).

Female Arterial Index

AGEW = −59.92 + 48.87⋅CIMmin + 2.4⋅AIx + 32.41⋅log (PWV) + 0.64⋅STENmax − 0.95⋅AIx⋅log (PWV) − 0.7⋅CIMmin⋅STENmax

Male Arterial Index:

AGEM = −0.86 + 46.68*CIMmin + 0.17*STENmax + 6.18* log (PWV).

Where:

*CIM*_*min*_–minimal thickness of the intima-media complex, in the left or right carotid, *AIx*–Augmentation Index (the degree of pressure rise in the artery after the return of the reflected wave; the difference of the dicrotic wave and the anacrota, divided by the central pulse pressure), *PWV*–pulse wave velocity, *STEN*_*max*_–Maximal of two stenosis values, on the left or on the right.

### Statistical processing

Statistical processing of the results was carried out using the STATISTICA v.12 program. To assess the statistical significance of the differences obtained, non-parametric criteria were used: Mann-Whitney for independent and Wilcoxon for dependent samples. The data in the table are presented in the form of median and interquartile ranges. The level of statistical significance was taken to correspond to p 0.05.

## Results

There were no cases of clinically significant abnormalities in the physical examination at any visit in any group. The average values of blood biochemical parameters in both groups did not differ at the beginning and at the end of the study.

According to the ultrasound examination of the carotid arteries in patients of the main group a statistically significant decrease in the minimum value of the thickness of the intima-media complex on the right or left from the initial was noted. No such changes were observed in the placebo group (Table 2).

**TABLE 2:** Results of ultrasound of carotid arteries and applanation tonometry.

Indicator	Comparison group		Main group	
	Before taking a placebo	After 90 days of taking a placebo	Before taking dietary supplements	After 90 days of taking dietary supplements
Minimum value of the thickness of the intima-media complex on the right or left (cIMT), mm	0.71	0.73	0.72	0,70 **
	0.66–0.80	0.65–0.80	0.68–0.81	0,64–0,73
Maximum carotid artery stenosis, right or left (STENmax), %	0	0	25.0	20
	0.0–25.0	0.0–20.0	0.00–25.0	0.00–25
Augmentation Index (AIx), %	27.0	28	29.0	27
	19.25–33.50	24.0–33.0	22.50–36.00	24.0–32.75
Pulse wave velocity, m/s	10.20	9.10	10.50	9.50*
	8.93–10.57	8.10–10.10	9.25–12.45	8.70–10.75

1. The data in the table are presented in the form of median and interquartile ranges.

2. The differences are statistically significant compared to the beginning of the study:

**- *р*< 0.001; * - *р*<0.01 (non-parametric criteria Wilcoxon for dependent samples).

A decrease in this parameter indicates favorable changes in the vascular wall, accompanied by an increase in the lumen of the carotid arteries and, accordingly, an improvement in the blood supply to the brain.

The maximum stenosis of the carotid artery, on the right or left in patients of both groups did not change statistically significantly during the treatment.

According to the results of applanation tonometry, it was revealed that while taking the studied dietary supplement, the pulse wave velocity decreased statistically significantly from the initial (Table 2).

There were no statistically significant changes in the placebo group. The decrease in pulse wave velocity reflects a decrease in arterial stiffness (a characteristic age-related change) and normalization of the reflected wave return velocity. These positive changes ultimately led to a decrease in the excess load on the left ventricle and an increase in perfusion pressure in the coronary arteries.

Table 3 shows the values of the biological age calculated from the data of the ultrasound of the carotid arteries and applanation tonometry.

**TABLE 3:** Biological age (years), calculated according to the data of the ultrasound of the carotid arteries and applanation tonometry.

Comparison group		Main group	
Before taking a placebo	After 90 days of taking a placebo	Before taking dietary supplements	After 90 days of taking dietary supplements
54.7	55.6	57.6	55.0**
50.5–61.2	50.6–61.0	50.5–61.6	48.7–59.4

1. The data in the table are presented in the form of median and interquartile ranges.

2. The differences are statistically significant compared to the beginning of the study:

**- *р*< 0.001 (nonparametric criteria Wilcoxon for dependent samples).

It decreased compared to the baseline in patients of the main group and did not change in patients from the comparison group.

## Discussion

Currently, there is a growing interest in biomarkers of biological age. Biological age is understood as a synthetic index consisting of one marker or a combination of several biological markers, which by itself or in combination with functional markers not only correlates with chronological age, but is also able to identify people “younger” or “older” than their chronological age in the same demographic group cohorts (Franceschi et al., 2018).

Carotid intima-media thickness (cIMT) is an established surrogate marker of atherosclerosis (Carpenter et al., 2016). This parameter is also associated with metabolic syndrome, insulin sensitivity and other age-related functional disorders (Lee et al., 2014; Roussel et al., 2016). It has been shown that the thickness of intima-media reliably predicts the progression of Alzheimer’s disease in general (Wang et al., 2016) and cognitive decline associated with Alzheimer’s disease, in particular (Buratti et al., 2015). In addition, revascularization improves cognitive functions, suggesting that the relationship between carotid artery stenosis and cognitive decline may be causal (Lal et al., 2011; Ortega et al., 2014). In addition, cIMT is largely associated with cardiovascular and overall mortality (Murakami et al., 2005).

Another predictor of cardiovascular diseases, the Augmentation index (AIx), is associated with the risk of symptomatic cardiovascular disease (Nurnberger et al., 2002). The pulse wave velocity in the aorta is a reliable predictor of future cardiovascular events and mortality from all causes—an increase in PWV in the aorta by 1 m/s corresponds to the risk adjusted for age, gender and risk factors for 14%, 15% and 15% of the total number of cardiovascular events, cardiovascular mortality, and mortality from all causes, respectively (Vlachopoulos et al., 2010). Thus, the aging of the arteries can be considered as a key factor in the overall aging process.

As a measure of biological age in this study we applied Artery Indices (Fedintsev et al., 2017). While creating this model, more than 80 hematological and functional health parameters were studied in a well-characterized cohort of patients for 2 years. Machine learning methods were applied to them, and the greatest predictive power was found for markers of arterial stiffness and artery wall thickness, which were combined into the Artery Indices model. Arterial Indices were determined by combining four functional indicators of cardiovascular health from the results of carotid duplex scanning and applanation tonometry. The advantage of this model is the non-invasiveness of measurements. The Artery Index was significantly higher in people with hypertension and type 2 diabetes than in healthy people that validates it is as a biological age predictor (Fedintsev et al., 2017).

It is assumed that the improvement and introduction of personalized non-drug interventions, including diet and exercise, are more likely to lead to a healthy aging of the population than new or repurposed drugs (Guerville et al., 2020). In addition, food supplements could help to improve certain parameters of a person’s quality of life: physical, mental, emotional, or social functioning.

It should be noted that the patients aged 40–60 years, whom we accepted into the study, were relatively healthy and did not need pharmacotherapy. However, it is known that atherosclerotic lesions and vascular stiffness at this age are already quite pronounced. Taking food additives with a high safety profile can help improve endothelial function and reduce biological age. This strategy can prolong the patient’s health and prevent the need for pharmacotherapy, which, despite its effectiveness, is often associated with undesirable drug reactions, individual intolerance, etc.

The dietary supplement contains a combination of terpenes of fir. It was revealed that in normal fibroblasts, terpenes induced genes for stress response, autophagy, apoptosis regulation, and tissue regeneration. The restoration of the expression level of some prolongevity genes after fir extract treatment was shown in senescent cells (Kudryavtseva et al., 2016). In subsequent preclinical studies on human fibroblasts, have shown that a substance containing fir terpenes exhibits antioxidant activity, induces autophagy, and affects aging-associated molecular pathways in the transcriptome and proteome (Kudryavtseva et al., 2016; Lipatova et al., 2021). Terpenoids exhibit many properties of potential geroprotectors that can effectively influence the mechanisms of aging and age-related diseases (Proshkina et al., 2020), including blood vessel endothelial disfunction.

In patients taking supplement for 3 months, there was a decrease in the minimum thickness of the intima-media complex on the right or left side, which was a manifestation of favorable changes in the vascular wall an increase in the lumen of the carotid arteries and, accordingly, an increase in the improvement of blood supply to the brain.

The decrease in pulse wave velocity in patients of the main group after a 3-month course of taking food additive reflects a decrease in arterial stiffness (a characteristic age-related change) and normalization of the rate of return of the reflected wave, which ultimately leads to a decrease in excessive load on the left ventricle and an increase in perfusion pressure in the coronary arteries.

In this work, the assessment of biological age and the effect of dietary supplements on it was carried out exclusively according to the parameters of the cardiovascular system. Therefore, it is natural that the positive effect obtained because of taking supplement was expressed in a decrease in biological age.

As mentioned above food additive contains polyunsaturated omega-3 fatty acids, vitamin E, limonene, Siberian fir terpenes, and placebo capsules contain flaxseed oil, which also contains unsaturated omega-3, 6 and 9 fatty acids and vitamin E. One would expect that patients in the placebo group would also experience positive effects, but we did not find statistically significant changes in the indicators characterizing the state of the vascular bed.

This suggests that the positive effect of terpenes on the stiffness of the vascular wall has been revealed.

A similar example is the European RISTOMED project, a multicenter open randomized study of the effect of a diet designed to meet the recommended daily requirement for nutrients, vitamins, and minerals in accordance with various cultural traditions, separately or with three nutraceuticals, including d-limonene, on inflammatory and metabolic markers. In healthy middle-aged people. It has been shown that the addition of d-limonene in the context of this dietary intervention can have a beneficial effect on the middle-aged, limiting the negative effects of chronic inflammation and improving the parameters of insulin resistance (Ostan et al., 2016).

In addition, it is likely that Siberian fir terpenes supplement, as well as any dietary additive, is characterized by a weak or moderate cumulative effect without a pronounced effect on laboratory and/or functional parameters of the body, which, in turn, characterizes the safety of food additives well. Siberian fir terpenes supplement has demonstrated a high safety profile. At the same time, additional research is needed to understand the detailed mechanisms of action of the composition of the substances that make up a supplement.

Thus, according to the instrumental method of examination (ultrasound examination and applanation tonometry), it was proved that taking the studied dietary supplement helps to improve blood supply to the brain. At the same time, it does not affect laboratory parameters, which confirms the safety of dietary supplements. The positive effect of the investigated dietary supplement on the condition of blood vessels was ultimately expressed in a statistically significant decrease in biological age, estimated by Artery Indexes model.

## Data Availability

The original contributions presented in the study are included in the article/Supplementary Material, further inquiries can be directed to the corresponding author.
