# Supplementary material for: A prospective randomized comparative placebo-controlled double-blind study in two groups to assess the effect of the use of biologically active additives with Siberian fir terpenes for the biological age of a person
Source: Front Pharmacol. 2023 Mar 2;14:1150504. doi: 10.3389/fphar.2023.1150504 (PMC10017525; doi:10.3389/fphar.2023.1150504)
Supplement: Supplementary file 1 [file DataSheet1.PDF]

Individual biochemical blood parameters in patients of both groups at the beginning and the end of the study

| Randomized n | Group | Gender | Age | Markers of carbohydrate metabolism and insulin resistance | | | | Markers of blood lipid profile | | | | | | Aminotransferases | | | |
|---|---|---|---|---|---|---|---|---|---|---|---|---|---|---|---|---|---|
| | | | | Fasting venous blood glucose, mmol/l | | Insulin, mkU/ml | | Triglycerides, mmol/l | | Cholesterol - HDL, mmol/l | | Cholesterol - LDL, mmol/l | | ALT, U/ml | | AST, U/ml | |
| | | | | Visit 1 | Visit 4 | Visit 1 | Visit 4 | Visit 1 | Visit 4 | Visit 1 | Visit 4 | Visit 1 | Visit 4 | Visit 1 | Visit 4 | Visit 1 | Visit 4 |
| 1 | treatment | fm | 41 | 5,26 | 4,98 | 11,70 | 2,95 | 1,01 | 1,05 | 1,99 | 1,72 | 3 | 3,21 | 19,7 | 17,4 | 16,5 | 17,3 |
| 2 | treatment | fm | 52 | 5,46 | 5,12 | 8,10 | 7,30 | 0,9 | 1,05 | 1,02 | 1,25 | 2,74 | 3,78 | 19,7 | 19,2 | 17,5 | 19,5 |
| 3 | treatment | m | 45 | 5,52 | 5,43 | 10,50 | 8,21 | 2,63 | 2,49 | 1,62 | 1,70 | 4,96 | 5,23 | 21,3 | 15,2 | 14,6 | 20,2 |
| 4 | treatment | fm | 44 | 5,17 | 4,99 | 17,30 | 10,50 | 0,82 | 1,21 | 1,81 | 1,81 | 3,45 | 3,99 | 32,7 | 31,5 | 21,7 | 17,7 |
| 5 | treatment | m | 48 | 6,58 | No data | 8,77 | 8,77 | No data | No data | No data | No data | 3,22 | No data | No data | No data | No data | No data |
| 6 | treatment | fm | 46 | 5,17 | 4,99 | 11,50 | 12,00 | 0,96 | 0,57 | 1,14 | 1,18 | 3,67 | 3,67 | 14,8 | 15,6 | 21,3 | 19,2 |
| 7 | treatment | fm | 49 | 6,11 | 5,41 | 12,40 | 6,15 | 1,40 | 1,83 | 1,42 | 1,51 | 5,15 | 5,15 | 15,9 | 21,1 | 15,3 | 22,1 |
| 10 | control | fm | 50 | 5,39 | 4,74 | 2,00 | 3,71 | 1,67 | 1,88 | 1,82 | 1,56 | 4,34 | 3,75 | 11,6 | 17,2 | 17,0 | 19,9 |
| 11 | treatment | fm | 51 | 5,12 | 4,81 | 6,81 | 2,00 | 0,63 | 0,70 | 1,56 | 1,63 | 3,21 | 3,04 | 18,4 | 23,0 | 23,4 | 29,9 |
| 12 | treatment | fm | 56 | 6,26 | 7,03 | 6,20 | 5,95 | 0,80 | 1,16 | 1,55 | 1,64 | 3,71 | 4,2 | 22,2 | 29,1 | 19,8 | 26,7 |
| 13 | control | fm | 56 | 4,97 | 4,96 | 15,90 | 11,20 | 1,98 | 2,24 | 1,55 | 1,45 | 4,7 | 4,27 | 32,9 | 30,6 | 24,2 | 24,0 |
| 15 | control | m | 42 | 5,25 | 5,04 | 8,42 | 18,60 | 1,52 | 1,54 | 1,27 | 1,14 | 2,49 | 2,26 | 21,9 | 22,2 | 19,9 | 19,2 |
| 16 | control | fm | 59 | 5,32 | 5,14 | 5,10 | 0,00 | 1,31 | No data | 1,56 | No data | 4,42 | No data | 29,0 | No data | 29,6 | No data |
| 17 | control | fm | 58 | 5,16 | 4,69 | 21,80 | 2,00 | 0,56 | 0,62 | 1,89 | 2,00 | 3,65 | 3,8 | 7,9 | 9,9 | 17,3 | 18,6 |
| 18 | treatment | m | 44 | 5,08 | 5,17 | 12,10 | 18,70 | 1,43 | 1,17 | 1,06 | 1,22 | 3,3 | 3,56 | 54,0 | 54,6 | 26,3 | 28,2 |
| 19 | control | fm | 42 | 5,21 | 4,85 | 2,00 | 10,00 | 3,25 | 3,27 | 0,91 | 1,06 | 2,97 | 3,1 | 28,8 | 29,2 | 20,4 | 20,9 |
| 20 | treatment | fm | 64 | 5,59 | 5,22 | 8,85 | 2,00 | 0,85 | 1,00 | 2,16 | 2,25 | 3,81 | 3,89 | 12,2 | 10,8 | 23,4 | 21,9 |
| 21 | control | fm | 43 | 5,07 | 5,46 | 9,82 | 12,80 | 0,94 | 0,62 | 1,54 | 1,65 | 3,57 | 4,03 | 54,2 | 42,0 | 29,6 | 25,5 |
| 24 | control | fm | 45 | 6,04 | 5,85 | 8,16 | 9,76 | 0,92 | 0,91 | 1,51 | 1,55 | 2,95 | 2,95 | 12,1 | 8,7 | 18,9 | 12,4 |
| 25 | treatment | m | 49 | 5,36 | 5,2 | 32,60 | 6,63 | 1,71 | 1,77 | 1,10 | 1,40 | 4,38 | 4,47 | 23,6 | 27,2 | 45,5 | 41,8 |
| 26 | treatment | fm | 53 | 5,00 | 4,88 | 5,59 | 25,80 | 1,91 | 2,67 | 1,99 | 2,05 | 6,01 | 5,07 | 13,0 | 11,1 | 15,7 | 15,7 |
| 28 | control | fm | 54 | 5,39 | 5,22 | 13,90 | 8,16 | 1,14 | 0,87 | 2,02 | 2,00 | 3,73 | 3,37 | 27,8 | 18,9 | 28,7 | 20,0 |

| | | | | | | | | | | | | | | | | | |
|---|---|---|---|---|---|---|---|---|---|---|---|---|---|---|---|---|---|
| 29 | control | m | 43 | 5,33 | 5,06 | 5,12 | 9,42 | 1,75 | 1,99 | 1,11 | 1,16 | 3,53 | 3,57 | 46,6 | 39,9 | 23,7 | 23,4 |
| 30 | control | fm | 41 | 4,61 | 4,98 | 13,70 | 10,60 | 0,84 | 0,79 | 1,59 | 1,68 | 2,9 | 3,27 | 18,0 | 17,4 | 18,0 | 17,7 |
| 31 | treatment | fm | 49 | 5,65 | 5,09 | 31,10 | 7,16 | 1,35 | 1,05 | 2,70 | 2,22 | 4,82 | 3,59 | 16,2 | 15,7 | 19,4 | 24,3 |
| 32 | treatment | fm | 6 | 5,52 | 5,3 | 13,20 | 30,80 | 0,92 | 1,00 | 1,89 | 1,94 | 3,68 | 4,69 | 40,4 | 16,2 | 38,1 | 31,4 |
| 34 | treatment | fm | 51 | 5,11 | 4,84 | 12,60 | 13,00 | 1,08 | 1,17 | 1,05 | 1,27 | 2,97 | 3,94 | 23,2 | 74,0 | 18,4 | 33,1 |
| 35 | treatment | m | 40 | 5,37 | 5,61 | 12,00 | 7,95 | 0,90 | 0,87 | 1,02 | 0,96 | 3,15 | 3,55 | 35,6 | 32,8 | 25,8 | 26,9 |
| 36 | control | fm | 46 | 5,59 | 5,37 | 6,62 | 7,22 | 1,63 | 1,18 | 1,66 | 1,52 | 4,54 | 4,3 | 14,5 | 11,2 | 18,7 | 16,2 |
| 37 | control | m | 41 | 5,07 | 4,86 | 18,20 | 6,55 | 1,81 | 1,59 | 1,13 | 1,23 | 4,14 | 4,39 | 20,6 | 39,9 | 20,2 | 26,7 |
| 38 | treatment | fm | 46 | 6,75 | 5,13 | 5,72 | 20,50 | 2,44 | 2,86 | 1,49 | 1,42 | 3,83 | 4,35 | 77,5 | 96,8 | 93,5 | 134,7 |
| 39 | treatment | fm | 42 | 5,45 | 5,36 | 10,80 | 2,00 | 0,67 | 0,76 | 1,59 | 1,54 | 3,72 | 3,65 | 3,8 | 4,0 | 10,9 | 11,7 |
| 40 | control | fm | 51 | 5,6 | 4,72 | 12,20 | 5,67 | 2,37 | 2,10 | 1,66 | 1,50 | 4,11 | 3,97 | 20,4 | 23,2 | 23,7 | 24,0 |
| 41 | control | fm | 51 | 4,8 | 4,82 | 4,97 | 2,36 | 0,72 | 0,63 | 2,08 | 2,05 | 3,92 | 3,29 | 26,0 | 23,0 | 23,4 | 27,7 |
| 42 | control | fm | 48 | 5,24 | 5,12 | 3,47 | 4,48 | 0,95 | 0,73 | 1,48 | 1,47 | 3,13 | 2,79 | 15,5 | 14,6 | 15,7 | 15,1 |
| 43 | treatment | fm | 64 | 4,94 | 4,77 | 7,98 | 3,95 | 1,14 | 1,23 | 1,50 | 1,60 | 3,26 | 3,2 | 17,0 | 15,2 | 16,8 | 16,2 |
| 44 | control | fm | 42 | 5,25 | 5,09 | 3,73 | 3,60 | 1,40 | 1,14 | 1,53 | 1,68 | 3,81 | 4,25 | 35,6 | 23,6 | 29,8 | 22,1 |
| 45 | control | fm | 42 | 5,11 | 6,45 | 6,80 | 11,70 | 0,55 | 0,71 | 1,60 | 1,66 | 3,19 | 3,93 | 15,1 | 17,1 | 22,0 | 21,5 |
| 46 | treatment | fm | 65 | 5,42 | 4,92 | 16,50 | 4,81 | 0,74 | 0,97 | 2,09 | 1,86 | 4,91 | 5,72 | 31,1 | 31,9 | 33,7 | 29,1 |
| 47 | control | fm | 40 | 5,06 | 4,99 | 5,17 | 12,30 | 0,91 | 1,05 | 1,05 | 1,12 | 2,13 | 2,2 | 25,7 | 23,2 | 21,0 | 18,9 |
| 48 | control | fm | 44 | 5,32 | 4,99 | 5,94 | 11,20 | 0,86 | 1,13 | 0,93 | 1,2 | 2,89 | 3,52 | 11,4 | 13,9 | 15,6 | 20,8 |
| 49 | control | fm | 65 | 4,22 | 4,97 | 13,00 | 2,85 | 1,39 | 1,21 | 1,54 | 1,51 | 4,44 | 4,13 | 30,5 | 19,4 | 28,5 | 23,1 |
| 50 | control | fm | 55 | 4,9 | 5,38 | 17,30 | 16,90 | 0,81 | 1,78 | 2,16 | 1,97 | 2,76 | 3,66 | 50,0 | 58,9 | 40,4 | 36,4 |
| 51 | control | fm | 50 | 5,15 | No data | 4,31 | | No data | No data | No data | No data | 3,35 | No data | No data | No data | No data | No data |
| 52 | treatment | fm | 48 | 4,41 | 5,2 | 9,29 | 6,63 | 1,55 | 1,77 | 1,6 | 1,4 | 4,95 | 4,47 | 29,8 | 27,2 | 29,1 | 41,8 |
| 53 | treatment | fm | 52 | 4,41 | 4,97 | 9,81 | 3,36 | 0,81 | 1,28 | 1,63 | 1,87 | 4,27 | 5,43 | 27,4 | 21,7 | 25,2 | 24,8 |
| 54 | control | fm | 52 | 5,22 | 5,26 | 18,50 | 5,32 | 0,72 | 0,87 | 1,51 | 1,85 | 3,47 | 3,76 | 16,5 | 22,5 | 21,1 | 27,0 |
| 55 | control | fm | 43 | 5,51 | 5,29 | 5,53 | 7,07 | 1,36 | 1,43 | 2,02 | 1,88 | 4,29 | 4,05 | 14,7 | 15,6 | 18,7 | 22,1 |
| 56 | treatment | fm | 61 | 4,75 | 4,98 | 9,24 | 4,08 | 2,39 | 1,44 | 1,43 | 1,38 | 2,95 | 3,34 | 22,1 | 19,7 | 20,4 | 19,8 |
| 57 | treatment | fm | 59 | 5,21 | 5 | 2,00 | 15,70 | 1,08 | 1,31 | 2,43 | 2,16 | 5,41 | 5,43 | 20,2 | 11,0 | 22,8 | 16,0 |
| 58 | treatment | fm | 40 | 5,28 | 5,24 | 14,30 | 2,88 | 2,38 | 1,52 | 1,86 | 1,78 | 3,43 | 4,33 | 14,3 | 31,1 | 17,7 | 32,9 |

| | | | | | | | | | | | | | | | | | |
|---|---|---|---|---|---|---|---|---|---|---|---|---|---|---|---|---|---|
| 59 | control | fm | 40 | 5,5 | 5,88 | 6,64 | 10,40 | 0,74 | 0,46 | 1,81 | 1,21 | 3,26 | 2,37 | 15,5 | 12,8 | 15,4 | 15,7 |
| 60 | control | fm | 42 | 5,22 | 4,37 | 5,34 | 2,35 | 0,81 | 0,85 | 1,92 | 1,56 | 4,7 | 4,5 | 17,0 | 13,7 | 19,5 | 15,2 |
| 61 | treatment | m | 51 | 5,02 | 5,51 | 8,17 | 6,95 | 2,80 | 2,98 | 2,03 | 1,94 | 5,79 | 5,98 | 39,5 | 38,4 | 25,9 | 23,2 |
| 62 | control | fm | 43 | 5,12 | 5,16 | 2,00 | 5,53 | 0,99 | 1,34 | 1,8 | 1,88 | 3,66 | 3,87 | 17,8 | 16,8 | 14,9 | 17,7 |
| 63 | control | fm | 50 | 5,15 | 4,72 | 5,78 | 2,00 | 0,48 | 0,49 | 1,96 | 1,64 | 2,81 | 3,02 | 12,8 | 11,4 | 23,6 | 19,8 |
| 64 | treatment | m | 52 | 5,62 | 5,64 | 6,20 | 3,11 | 0,95 | 1,00 | 1,7 | 1,75 | 4,18 | 3,91 | 36,1 | 31,6 | 32,3 | 28,9 |
| 65 | treatment | m | 64 | 5,46 | 5,8 | 6,89 | 7,10 | 2,26 | 2,22 | 1,77 | 1,84 | 5,48 | 5,18 | 18,4 | 18,7 | 18,7 | 19,0 |
| 66 | control | fm | 45 | 6,3 | 5,94 | 2,94 | 6,67 | 1,06 | 1,00 | 1,71 | 1,93 | 4,87 | 5,11 | 13,9 | 21,2 | 17,8 | 21,1 |
| 67 | control | fm | 48 | 5,46 | 5,1 | 9,21 | 2,85 | 1,64 | 3,24 | 1,54 | 1,74 | 4,76 | 5,01 | 56,1 | 77,6 | 34,4 | 40,9 |
| 68 | treatment | fm | 59 | 6,35 | 5,58 | 11,70 | 5,08 | 2,24 | 1,48 | 1,03 | 1,32 | 2,91 | 3,70 | 11,0 | 12,2 | 16,8 | 18,6 |
